# A survey of Ethiopian physicians’ experiences of bedside rationing: extensive resource scarcity, tough decisions and adverse consequences

**DOI:** 10.1186/s12913-015-1131-6

**Published:** 2015-10-14

**Authors:** Frehiwot Berhane Defaye, Dawit Desalegn, Marion Danis, Samia Hurst, Yemane Berhane, Ole Frithjof Norheim, Ingrid Miljeteig

**Affiliations:** Research Group in Global Health Priorities, Department of Global Public Health and Primary Care, University of Bergen, Kalfarveien 21, 5018 Bergen, Norway; College of Health Science, Addis Ababa University, Addis Ababa, Ethiopia; Department of Bioethics, National Institute of Health, Bethesda, USA; Institute for Ethics, History, and the Humanities, Geneva University Medical School, Geneva, Switzerland; Addis Continental Institute of Public Health, Addis Ababa, Ethiopia; Department of Research and Development, Helse Bergen Health Trust, Bergen, Norway

**Keywords:** Ethics, Clinical, Allocation of health care resources, Health provider perspective, Bedside rationing, Ethiopia

## Abstract

**Background:**

Resource scarcity in health care is a universal challenge. In high-income settings, bedside rationing is commonly discussed and debated as a means to addressing scarcity. However, little is known about physicians’ experiences in resource-limited contexts in low- income countries. Here we describe physicians’ experiences regarding scarcity of resources, bedside rationing, use of various strategies to save resources, and perceptions of the consequences of rationing in Ethiopia.

**Methods:**

A national survey was conducted amongst physicians from 49 public hospitals using stratified, multi-stage sampling in six regions. All physicians in the selected hospitals were invited to respond to a self-administered questionnaire. Data were weighted and analyzed using descriptive statistics.

**Results:**

In total, 587 physicians responded (91 % response rate). The majority had experienced system-wide shortages of various types of medical services. The services most frequently reported to be in short supply, either daily or weekly, were access to surgery, specialist and intensive care units, drug prescriptions and admission to hospital (52, 49, 46, 47 and 46 % respectively). The most common rationing strategies used daily or weekly were limiting laboratory tests, hospital drugs, radiological investigations and providing second best treatment (47, 47, 47 and 39 % respectively). Availability of institutional or national guidelines for whom to see and treat first was lacking. Almost all respondents had witnessed different adverse consequences of resource scarcity; 54 % reported seeing patients who, in their estimation, had died due to resource scarcity. Almost 9 out of 10 physicians were so troubled by limited resources that they often regretted their choice of profession.

**Conclusion:**

This study provides the first glimpses of the untold story of resource shortage and bedside rationing in Ethiopia. Physicians encounter numerous dilemmas due to resource scarcity, and they report they lack adequate guidance for how to handle them. The consequences for patients and the professionals are substantial.

**Electronic supplementary material:**

The online version of this article (doi:10.1186/s12913-015-1131-6) contains supplementary material, which is available to authorized users.

## Background

The practice of medicine is resource intensive and there is always a gap between demand and supply. All health care systems therefore have to set priorities [1]. While politicians and policymakers make decisions for large patient groups and future patients, clinicians have to occasionally deny a *person* in front of them beneficial, and in some cases, vital treatment [2]. In so doing, clinicians must balance the two roles of being patient advocates and gatekeepers of resources [3, 4].

We define priority setting in health care as the ranking of health services and the ranking of recipients of these services. Priorities are often set through a process of decision-making. The ranking of services or patients can be systematic, partly arbitrary, or ad hoc and is typically a result of planned policies, financing mechanisms, historical budgets, legal regulations, the interests of health professionals, the influence of patient organizations, and public opinion [5, 6]. We define rationing as withholding of health services that could be of benefit on the grounds of resource scarcity [7, 8]. Resources should be understood broadly to include health personnel, time, equipment, infrastructure, medicines, beds, operating rooms, and money. In this paper we distinguish between system-wide rationing and bedside rationing. System-wide rationing is the withholding of beneficial health services for groups of people, and bedside rationing is the withholding of beneficial health services for individuals.

Clinical priority setting and bedside rationing by physicians have mainly been explored in studies from high-income settings. In a multi-country study of values at the bedside, Hurst et al. found that European physicians face resource scarcity and practice bedside rationing [8, 9]. In a systematic review of bedside rationing in high-income countries, Strech et al report that the percentages of respondents willing to accept rationing ranged from 94 to 9 % in various studies. That review illustrates the ambivalence of physicians towards rationing, but also points out how the context in which rationing occurs and the wording of survey questions influence physician attitudes [10]. A review of qualitative studies on bedside resource allocations also show how physicians’ rationing behavior is highly variable, strongly influenced by context-related factors, and consists mainly of implicit rationing strategies. Torn between patient advocacy and the obligation to contain costs, physicians experience various role conflicts [11].

Turning to low-income countries, there are studies on how to set priorities at the national level, while empirical studies of bedside rationing are scant [12]. Kapiriri et al studied priority setting in a hospital in Uganda and found that the priority-setting decisions did not satisfy the conditions of procedural fairness when evaluating them against a widely used framework– ‘accountability for reasonableness [13]. Johansson et al described bedside rationing in provision of anti-retro-viral treatment in Tanzania, where a first-come, first–served strategy was dominant [14]. In a study of hospital care of neonates in India, Miljeteig et al describe how doctors experienced lack of basic equipment and resources forcing them to choose between which patient to treat or let die [15] and how factors external to a newborn’s health status (such as household poverty) influence physicians’ decisions about treatment [16, 17]. We have not been able to identify any systematic, nation-wide, empirical studies of bedside rationing in low or middle-income countries.

The aim of this study in Ethiopia was to provide a systematic description of physicians’ experiences of resource scarcity, the bedside rationing strategies they used in public hospitals, and their perception of the consequences of rationing for patients and themselves.

## Methods

### Study design, partisipants and setting

This was a nation-wide, cross-sectional survey of all categories of physicians working in public hospitals in Ethiopia. Ethiopia is the second most populous country in Africa with geographic, socio-economic, cultural and religious diversity. Though currently the country is undergoing rapid development, there is a substantial gap between demand and supply of health care because of resource scarcity, poverty and high burden of disease [18]. Table 1 depicts some key indicators relevant to understand health development in Ethiopia.

**Table 1:** Demographic Health and Development indicators of Ethiopia

Total population	95.9 million^a^
Life expectancy at birth (years)	62^a^
Total fertility rate	4.1^a^
Maternal mortality ratio (per 100,000 live births)	420^a^
Infant mortality rate (per 1000 live births)	50^a^
Under-5 mortality rate (per 1000 live births)	68^a^
Stunting in children under 5 years of age	40.1 %^b^
Hospital to population ratio	1:564173^a^
Number of hospitals (by levels/types)	125^a^
Physicians (GPs and Specialist) to population ratio	1:32132^a^
Total number of general practitioners	1213^a^
Total number of Specialists	331^a^
Health expenditure as % of GDP	4.7^c^
Per capital total expenditure on health	US$ 20.77^d^
Out of pocket payments (as % of total health expenditure)	34 %^d^

Sources:

^*a*^ *Federal Democratic Republic of Ethiopia Ministry of Health, Ministry of Health Health and Health Related Indicators November 2014* http://www.moh.gov.et/documents/26765/0/Health+and+Health+Related+Indicators+2005+E.C/1b5b2a9f-a960-4024-8d92-519195364023?version=1.0

^*b*^ *Central Statistics Agency [Ethiopia] Mini Demographic and Health Survey August 2014* http://www.unicef.org/ethiopia/Mini_DHS_2014__Final_Report.pdf

^*c*^ *UNDP –* hdr.undp.org/en/data

^*d*^ *Federal Democratic Republic of Ethiopia Ministry of Health, Fifth National Health Accounts, 2010/2011. Addis Ababa. Ethiopia,* https://www.hfgproject.org/wp-content/uploads/2014/04/Ethiopia-NHA-Findings-Briefing-Notes.pdf

### Sampling procedure

To obtain a representative sample of categories of regions (urban, rural and pastoralist), we randomly selected two regions from each category (six of 11 regions). The region of Addis Ababa was purposively included as most specialized physicians work in the capital and we wanted to make sure to get their responses. We then applied probability sampling, and weighting was done according to the numbers of hospitals in each region. Accordingly, we selected 49 hospitals. At each hospital , we included all physicians working there at the time of the study. We excluded physicians that had less than 1-year working experience.

### Data collection

As this study was the first of its kind in a low-income setting, we developed our questionnaire from a previously validated tool used in the US and four European countries [19, 20]. The questionnaire was contextualized to the Ethiopian setting through cognitive testing, pilot testing, and reformulation of unfamiliar terms, inclusion of context specific issues, and preferences of physicians on data collection modality, language and timing.

Physicians were recruited from July to November in 2013 by one of the authors (FBD) at the end of their morning meetings or at their work place and were given written information explaining the aims of the study, a consent form to be signed separately, and an envelope with the self-administered questionnaire to be returned anonymously.

### The questionnaire

The questionnaire addressed various ethical dilemmas faced by physicians in Ethiopia; the majority of the questions focused on experiences with resource scarcity and the perceived consequences; unavailable and rationed services, criteria used and strategies to handle limitations and protect against catastrophic health expenditures (the questionnaire is available as Additional file 1). This paper reports the results concerning respondents’ bedside experiences of scarcity, rationing, and their consequences.

### Statistical analysis

Data were coded, entered using EPI INFO, cleaned and weighted according to sample size using Stata13.1 statistical software. Responses were analyzed using descriptive statistics.

### Ethical considerations

The research was conducted in accordance with the principles for medical research as described by the Helsinki Declaration. There were no known risks for the participants, and they did not directly benefit from participation in this study. All participants gave written informed consent. Data were handled and analyzed anonymously. Ethical approval was obtained from the IRB of Addis Ababa University College of Health Sciences and US National Institute of Health and Development, and exempted by the Norwegian Regional Committee for Medical Research Ethics.

## Results

### Respondents

Of the 640 distributed questionnaires, 587 responded (response rate 91 %). Physicians with less than 1-year of service were excluded and final analysis was done on 565 respondents. According to the 2012 Health and Health Related Indicators from the Ethiopian Ministry of Health, there were approximate 1544 practicing physicians (938 GPs and 606 Specialists) in Ethiopia and 116 hospitals in 2012 [21]. Our survey thus included about 38 % of all physicians and 42 % of the total number of hospitals in the country.

Most respondents were men (78 %) and young (mean age was 31.1, median age 28 years), and had less than 6 years of service (ranging from 1–32 years) (Table 2). Half of them were general practitioners, while approximately ¼ were specialists and ¼ residents.

**Table 2:** Respondents Characteristics

Characteristics^a^	Percent	Total N
Gender:		
Women	21	563
Men	79	
Mean Age	31 (23–64) SD = 8.1	555
Age group:		
< 31	68	555
31–40	21	
41–50	9	
> 50	3	
Undergraduate medical training:		
Ethiopia	94	551
Abroad	6	
Postgraduate medical training:		
Ethiopia	94	278
Abroad	6	
Mean service year	6 (1–32) SD = 6.96	557
Years in practice:		
1–5	70	540
6–10	15	
11–20	9	
> =21	6	
Professional status:		
GPs	49	556
Specialists	24	
Residents	27	
Have private practice	38	565
Average work hour/week:		
Government	46 (SD = 3.1)	525
Private	20 (SD = 1.3)	28
Average number of patients/week (in government hospital)	10–600 (SD = 13.5)	525
Involvement in medical academics (yes)	72	518
Involved as:		
Instructor	53	413
Resident	36	
Researcher	5	
Others	6	
Involvement in planning and decision making:		
Yes	28 (150)	559
No	409 (71)	

^a^All respondents were government employed

Analysis done on valid N, excluding missing and not applicable

One out of five doctors reported having additional private practice. Less than one third (28 %) of the respondents reported participating in planning and decision-making in their hospitals.

### Frequency of rationing dilemmas encountered

Respondents frequently reported encountering rationing dilemmas (Table 3). Almost all claimed that scarcity of resources required them to make difficult choices (99 %). Reallocation of resources, by restricting treatment to one patient for the benefit of others who could gain more benefits, was reported by 66 % of responding physicians.

**Table 3:** Rationing Dilemmas Encountered by Physicians

Rationing dilemmas	Often (%)	Sometimes (%)	Rarely (%)	Never (%)	Total N
Restricting treatment to a patient to give those resources to someone who could benefit more	18	33	15	34	538
Felt that the patients need of treatment was not in agreement with the family need or welfare	29	49	13	9	529
Limitation of resources required me to make difficult choice	5	39	56	1	551
There was significant disagreement among health personnel on continuing treatment of the patient due to lack of resources	30	39	19	11	541

We asked about indications of rationing and found that 89 % of the physicians had experienced significant disagreement amongst health care personnel about continuing or not continuing treatment for patients due to lack of resources. Patient’s inability to pay for services had led 95 % of the participating physicians to sometimes forgo the preferred course of treatment for their patients.

### Scarcity

**Fig. 1:**
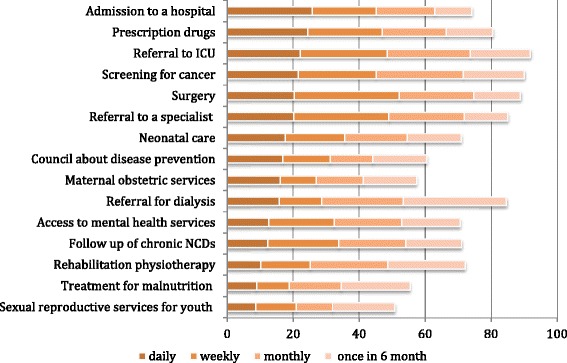
Experience of Scarcity by Physicians. In response to the question: “During the last 2 years, how often were you unable to obtain the following services for your patients when you thought they were necessary?” The various medical interventions are presented from the top to the bottom of the graph according to the likely frequency of daily demand for these interventions

System-wide rationing is experienced in all settings (Fig. 1).

Physicians were asked a series of questions containing the following stem: “During the last 2 years, how often (daily, weekly, monthly and 6 monthy) were you unable to obtain the following services for your patients when you thought they were necessary?” The frequency of scarcity varied between 51 and 92 % for different services. Limited availability of referrals to surgery, specialists and ICU beds, prescriptions of drugs and hospital beds were most frequently experienced on a daily or weekly basis. Access to sexual reproductive health services for young people, treatment for malnutrition, and access to rehabilitation therapy and follow up for chronic non-communicable diseases were less often rationed.

### Rationing strategies

**Fig. 2:**
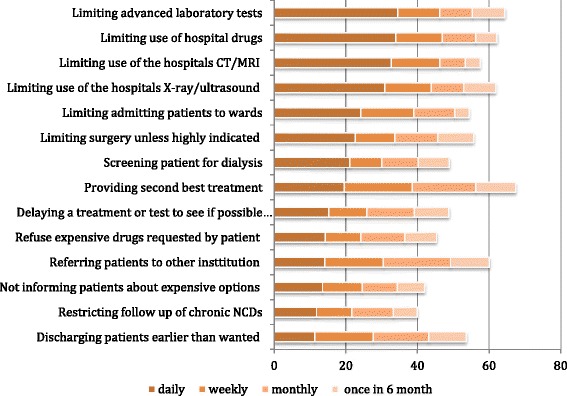
Strategies Used by Physicians to Ration by the Bedside. In response to the question: “Consider a situation when expenses must be covered by the institution you work in. During the last 12 months, how often did you try to save costs for your institution by … The various medical interventions are presented from the top to the bottom of the graph according to the likely frequency of daily demand for these interventions

Physicians use different bedside rationing strategies (Fig. 2).

To identify different mechanisms of bedside rationing, we asked our respondents a series of questions containing the following stem: “During the last 12 months, how often (daily, weekly, monthly, 6 monthly) did you try to save costs for your institution by …”. The five most commonly reported strategies for rationing to save institutional costs were limiting advanced laboratory tests, limiting the use of hospital drugs; limiting use of the hospital’s x-ray, ultrasound, and CT/MRI equipment; providing second best treatments; and referral of patients to other institutions (strategies used daily or weekly by 39–47 % of our respondents). We also asked about other rationing strategies, and 41 % of the respondents agreed that they use a first come first served strategy to allocate scarce resources.

### Availability of guidelines

We were interested in whether physicians had tools to guide them in bedside rationing decisions, and we therefore asked about whether they have guidelines that could be applied. We found that there is a perceived inadequacy of guidelines on how to exercise decision-making to prioritize patients in need of various types of clinical care (Table 4).

**Table 4:** Availability of Guidelines

Guidelines for:	Yes (%)	No (%)	Do not know (%)	Total N
Which patient to see first	32	57	11	530
Which treatment the patients receive?	39	55	6	530
Which patients are taken to admission first?	33	59	9	532
Which patients are admitted to the ICU?	23	63	14	527
Which patients are taken to the OR first?	27	59	15	525

**Table 5:** Physicians’ Experiences Regarding Resource Scarcity

	Daily (%)	Weekly (%)	Monthly (%)	6 monthly (%)	Never (%)	Total N
Felt under pressure to deny an expensive intervention that I thought was indicated because of lack of resources	31	23	20	15	12	533
Encountered patients who have problems that cannot be treated because they cannot afford the treatment	35	28	24	10	4	539
Seen a situation where a patient suffered adverse consequences as a result of limited resources in the health care system	29	23	25	20	3	538
Been so troubled by limited resources that I regretted my choice of profession	36	23	15	15	12	538

More than 60 % said that they did not have guidelines in their institutions for which patients are admitted to the ICU. More than 55 % said they do not have institutional or national guideline that can help to decide which patients to admit first, which patients to initially take to an operating room, or what kind of treatment to provide.

### Adverse effects of scarcity and regrets about taking up the profession

We were interested in what kind of impact scarcity has and how this experience affects physicians’ work satisfaction (Table 5).

For the question “Have you seen a situation where a patient suffered adverse consequences as a result of limited resources in the health care system?” almost all (97 %) said yes, of which 29 %, 23 %, 25 % did so on a daily, weekly and monthly basis respectively. Among the consequences they have seen, 54 % had encountered deaths, 19 % acute life threatening events, while 15 % had encountered permanent or temporary disabilities that they attributed to scarcity of resources. Over all 88 % (36 % on a daily and 23 % on a weekly basis) reported that they had been so troubled by limited resources that they regretted having chosen their profession.

## Discussion

Our predominant findings are that scarcity-related dilemmas are frequently encountered by physicians in Ethiopia and that system-wide and bedside rationing are occurring in all settings. Physicians report using different strategies to ration and they lack guidelines on how to prioritize patients in need of care. Most of the responding physicians have encountered serious and even life-threatening adverse consequences of rationing. As a result of resource scarcity, a large majority has some regrets about choosing their profession.

Our finding that specialized and often costly services – such as intensive care, screening for cancer, admission to surgery, referral to specialist, referral for dialysis, access to prescription drugs, and admission to hospital – are scarce and rationing at the bedside is not unexpected, given the high priority assigned to primary care services in Ethiopia [18]. At the time of our survey the number of fully equipped intensive care beds in public hospitals was not more than 30, the number of surgeons working in the public sector about 60, the number of public dialysis centers for the whole country was only two, and the list of essential drugs was limited in Ethiopia. Ethiopian health planners have set clear priorities through the definition of essential health care and have done a lot to prioritize primary health care services, especially through the use of Health Extension Workers [22]. This implies that more costly and more specialized services like ICUs, surgery, general hospital services are ranked lower, and assigned lower priority for public funding. Now, Ethiopia is aiming to be a middle-income country by 2025. Studies from other middle-income countries show that economic development and decrease in mortality lead to higher expectations and demands for advanced care [23]. The expanding private market providing these services in urban areas like Addis Ababa and the willingness to go abroad to get access to advanced treatment suggest that the same pressures for costly and medical services will arise in Ethiopia in the years to come [24]. There is clearly a need for more resources, and it is likely that efficient, but also more costly treatments will be included among essential services in the next 5-year plan. There is also a need for understanding how current plans and priorities at macro- and meso -level translate into rationing dilemmas for physicians, and how those decisions can be made more efficiently and fairly [25].

Physicians often use implicit rationing strategies [10, 11, 21]. In our study we find that a first-come, first-served strategy was often used. Studies show how this strategy can be unfair [26]. Within the field of priority-setting ethics, there is common agreement about and recommendations regarding more openness and transparency about rationing because this can identify resource gaps and evidence gaps, improve social learning, and create opportunities for public involvement [27]. A valuable component of such priority setting is the development, implementation, and use of guidelines [28]. Studies on antiretroviral treatment guidelines indicate that they are well implemented and used in low-income countries and can have substantial impact on the distribution of health outcomes [14, 29]. Kapiriri et al suggest that resource-sensitive clinical guidelines could help physicians in LICs make difficult decisions [30]. To our knowledge, specific ethical guidelines for clinical rationing have not been made at a national level in Ethiopia. Our findings indicate that guidelines for allocation of clinical interventions among patients were not widely available. Therefore decisions could vary across professionals, programs and institutions. Ethiopia has developed clinical guidelines in many areas and this important work could be further strengthened – especially in areas where rationing dilemmas are widespread.

Even though resource scarcity may be perceived as a normal phenomenon in a low-income country, our results indicate that our respondents experience the adverse consequences of rationing as quite stressful. In addition to being overwhelmed by their workload due to a physician-to-population ratio of about 1:32,000, [21] Ethiopian physicians have to make tough decisions including limiting diagnostic or therapeutic interventions for some patients to provide them to others who may need them more. There are probably many other factors aside from “being so troubled by lack of resources” that lead to the high proportion of physician regretting their profession. Patients and community dissatisfaction and complaints, high workload, low salary, decreasing status and high private demands can aggravate the stress level, and regret [31]. Nevertheless, it is likely that not being able to do what they know ought to be done can play a role in work dissatisfaction. Our study participants are young; many have recently graduated from universities where they are trained to provide the best treatment. Many of them have few colleagues to debrief or talk with in breaks, and only 1/3 participate in the planning and administration of the hospital in which they work. Physicians have an important role as both the patient advocate and gatekeeper of resources. They could be trained to understand why and how priority setting happens at all levels, take an active role in priority setting, and play an important role in planning and decision-making at all levels.

### Strengths and limitations

This study provides new information on the highly relevant and yet sensitive issue of allocation of scarce resources at the clinical level. Our study includes all categories of physicians (approximately 38 % of all physicians in Ethiopia) working at all levels of the 49 hospitals in six randomly selected regions out of 11 in the country. We also achieved a high response rate (91 %). The findings in this survey should therefore be representative for the whole country, although some of the most underserved regions were not selected during our randomization. Experiences of rationing would probably be even higher in those regions. Despite this limitation, we believe the findings could provide lessons for other countries and health systems in similar settings.

Our study also had other limitations, and conclusions should therefore be drawn with some caution. Though our respondents were well educated and we tried to simplify the questionnaire, some words and concepts were unfamiliar because they had never been exposed to such a study before. The survey was quite comprehensive and cognitively demanding. Moreover, as is the case with all self-administered questionnaires, missing data are inevitable, but in our case this was not more than 5 %.

Another limitation reflects the fact that physicians are a small part of the healthcare workforce in Ethiopia. Mid-level health workers such as health officers, midwives and nurses perform most of the functions in primary care and should therefore also be studied. Our informants were working primarily in the public system. The private health care system provides a growing proportion of the health services provided in Ethiopia. While our survey provides valuable information, a full picture of limited resources and bedside rationing would also include their perspectives.

## Conclusions

This study provides the first glimpse of the untold story of scarcity and bedside rationing in a low-income country such as Ethiopia. Physicians encounter numerous dilemmas due to limited resources. System-wide and bedside rationing is highly prevalent in Ethiopia, and physicians adopt different rationing strategies without substantial guidelines. There is clearly a need for a more systematic approach to priority-setting and bedside rationing.

## Additional file

Additional file 1:
**Questionaire used in the study "Values at the bedside Ethiopia". **(PDF 559 kb)
