# Supplementary material for: A survey of Ethiopian physicians’ experiences of bedside rationing: extensive resource scarcity, tough decisions and adverse consequences
Source: BMC Health Serv Res. 2015 Oct 14;15:467. doi: 10.1186/s12913-015-1131-6 (PMC4607248; doi:10.1186/s12913-015-1131-6)
Supplement: Additional file 1: — Questionaire used in the study "Values at the bedside Ethiopia". (PDF 559 kb) [file 12913_2015_1131_MOESM1_ESM.pdf]

Values at the Bedside in Ethiopia

A Survey of Physicians Regarding Ethical Dilemmas in Clinical Practice

Thank you for your participation! Your contribution will be highly valued, as getting information from physicians working "on the ground" with patients is crucial in this first study of medical ethical dilemmas in Ethiopia. The data from this survey will help us in making teaching and training in medical ethics for students and clinicians context adjusted and relevant. It will also be useful for evidence based policy and practice in medical ethics. Since there are important differences between health care levels, some questions may seem not applicable to you, but please answer as best you can based upon your situation. We value your opinions, and hope you help us with filling out the whole questionnaire.

Part I: Background

1. Gender: 1. Male 2. Female

2. Age: ________Years

3. Where did you have your medical training?

    1. Undergraduate: Country _______________Medical school _________________

    2. Post graduate: Country _______________Medical school _________________

4. Since graduating, as MD, how many years have you been practicing medicine?_____ years

5. Are you currently working as

    1. GP 3. Resident in (please specify)________

    2. Specialist in (please specify)________ 4. Other (please specify)__________

6. Where do you practice as a doctor? (you can check more than one if applicable)?

    1. Government institution 4. Private non profit institution

    2. Private for profit institution 5. Own private institution

    3. Private wing in a government facility 6. Others__________

7. On average how many hours/ week do you spend in

    1. Government institution ____ 2. Private wing______ 3. Private institution_____

8. On average how many patients do you see in a week (both inpatient and outpatient)? ___________

9. If you are involved in medical academics, what is your position (-s)?

    1.Instructor 2. Resident 3.Researcher 4.Others__________ 5. Not involved

10. Do you participate in any decisions on where the resources are spent/allocated like in planning and budgeting in your health care department/facility?

    1. Yes 2. No

Part II: Ethical Dilemmas

11. Below is a list of situations where medical decision-making can be difficult. **In the last two years** how often have you been in the following situations?

	**Often**	**Some-times**	**Rarely**	**Never**	**Not applic-able**
a) You worried if you were helping or hurting the patient with the interventions					
b) You cared for a terminally ill patient and the question on when to stop treatment or a "Do not resuscitate" order came up					
c) You were withholding (not starting) potentially life prolonging treatment to a seriously sick patient to prevent prolonging death and suffering					
d) You were withdrawing (removing) potentially life prolonging treatment to a seriously sick patient to prevent death and suffering.					
e) You felt you were over-treating patients, i.e. providing treatment or diagnostic tests they could not benefit from					
f) You were restricting treatment to a patient to give those resources to someone who could benefit more (i.e. hospital bed, ventilator, medication)					
g) You felt that the patients need of treatment was not in agreement with the patient's family needs or welfare					
h) The preferred course of treatment was not pursued because of a patient's ability to pay					
i) Limitation of resources required you to make a difficult choice					
j) Your preferred course of treatment conflicted with institutional policies, professionalcodes of ethics or laws					
k) There was significant disagreement among health care personnel on continuing treatment of the patient due to lack of resources					
l) There was significant disagreement among family members on continuing treatment of the patient					
m You were in doubt if a diagnosis should be disclosed to the patient					
n) You were in doubt to disclose sexual reproductive health problem of an adolescent under the age of consent to a parent or a guardian					
o) You were in doubt whether to reveal adult patient's medical information to the family					
p) A patient's cultural or religious views conflicted with your proposed course of treatment					
q) You were asked to help a patient to have a comfortable death or to take their own life					
r) You cared for adult patients that were not in a state to make a decision for themselves (like unconscious/ disabled), and you had to decide for them					
s) You were in doubt when asked to perform an abortion or refer a woman for abortion					
t) You were in doubt whether to provide sexual reproductive health services (i.e. contraceptives, abortion,) to someone under the age of consent					
u) You witnessed that a colleague was not acting according to professional standards(like not being honest, fair, responsible and respectful)					
v) I came across colleagues that compromise quality of care in the public system for the sake of their private practice					
w) I came across colleagues not providing appropriate care because of inadequate medical knowledge and skills					
x) I felt conflicted between my obligations to the patients in the public hospital and to my patients in private practice					

12. If you have experienced any of the situations listed in the previous table, or any other striking ethical dilemma, can you please describe a dilemma you have encountered in your own words? If you do not have enough room below, please continue on the back of the questionnaire. (If you want to respond to this question in Amharic please do so)

13. Is the situation (-s) you have described above common where you work?

1. Yes, quite common
2. Happens sometimes
3. Happens rarely

Part III:Training and tools in medical ethics

14. Have you had medical ethics courses/lectures in medical school?
    1. Yes 2. No

15. Have you taken course in medical ethics after you graduated?
    1. Yes 2. No
16. If you had ethics training, how did you find it? (more than one answer is possible)
    1. Adequate 2. Not adequate 3. Not adjusted for our setting 4. Others____________ please specify

17. In general, how confident do you feel about your competence in handling ethical issues related to your practice?
    1. Very confident 2. Moderately confident 3.Not very confident 4. Not confident at all

18. Do you have any of the following ethics support services?

	Yes	No	Do not know
a)Courses or training programs aimed to increase health personnel's ethical competence			
b) Ethical guidelines for handling ethical dilemmas in clinical work			
c) Regular staff meetings where clinical ethics/ethical cases are the agenda			
d) Routines of debriefing among colleagues when ethical cases are experienced			
e) Mechanisms for contacting people who are specially trained in medical ethics			
f) Mechanisms for contacting people who are specially trained in law and regulations			
g) Mechanisms of creating ethical standards/guidelines at various departments			
h) Committees that can discuss/ give advice in concrete ethical dilemmas			
i) Others – please specify:			

19. What kind of ethics support do you think would be useful in Ethiopian setting?

	Yes	No	Do not know
a) Clinical ethics course in medical school			
b) Courses in clinical ethics obligatory to attend during specialization			
c) Teaching material/books in medical ethics developed for Ethiopian context			
c) Courses or training programs aimed to increase health personnel's ethical competence			
e) Ethical guidelines for handling ethical dilemmas in clinical work			
f) Regular staff meetings where clinical ethics/ethical cases are the agenda			
g) Routines of debriefing among colleagues when ethical cases are experienced			
h) Mechanisms for contacting people who are specially trained in medical ethics			
i) Mechanisms for contacting people who are specially trained in law and regulations			
j) Mechanisms of creating ethical standards/guidelines at various departments			
k) Committees that can discuss/ give advice in concrete ethical dilemmas			
l)Others – please specify:			

Part IV: Resources allocation

In the last two years, how often;

	Daily	Weekly	Monthly	Once in six months	Never	Not applic able
20)Have you been so troubled by limited resources that you regretted that you choose your profession						
21)Have you felt under pressure to deny an expensive intervention that you thought was indicated. because of lack of resources						
22)Have you felt under pressure by the owner of the Private institution to order an expensive intervention that you thought could have been managed with less cost						
23)Have you encountered patients who have problems that cannot be treated because they cannot afford the treatment						
24)Have you seen a situation where a patient suffered adverse consequences as a result of limited resources in the health care system						

25. What is the most severe adverse consequence you have seen as a result of limited resources in the health care system? (Check only one)
    1. Inconvenience 2. Temporary disability 3. Permanent disability
    4. An acute life-threatening event 5. Death 6. None

26. During the last two years, how often were you unable to obtain the following services for your patients when you thought they were necessary (this includes unacceptable waiting times)?

	Daily	Weekly	Monthly	Once in 6 months	Never	Not applic able
a) Surgery						
b) Referral to a specialist						
c) Admission to a hospital						
d) Access to mental health services						
e) Follow up of chronic non communicable conditions like CVDs/diabetes/cancer						
f) Screening for cancer						
g) Prescription drugs						
h) Referral to ICU						
i) Referral for dialysis						
j) An ethics consultation						
k) Maternal Obstetric services						
l) Neonatal care						
m)Treatment for malnutrition						
n)Sexual reproductive services for young people (contraception, abortion, care for rape victims etc)						
o)Rehabilitation/physiotherapy						
p) Council about disease prevention (e.g. smokingcessation, safe sex, hand washing etc.)						
q)Are there other relevant services in short supply, please specify						

27. At your institution, do you have any of the following?

	Yes	No	Do not know
a) Guidelines for which patients are seen first?			
b) Guidelines for which treatment the patients receive?			
c) Guidelines for which patients are taken to admission first?			
d) Guidelines for which patients are admitted to the ICU?			
e) Guidelines for which patients are taken to the OR first?			
f) A first come, first served strategy to distribute beds/surgery/limited resources?			

28. Consider a situation when expenses must be covered by the institution you work in. During the **last twelve months**, how often did you try to save costs for your institution by**:**

	Daily	Weekly	Monthly	Once in 6 months	Never	Not applicable
a)Limiting use of hospital drugs						
b) Limiting admitting patients to wards/ ICU						
c)Limiting surgery unless highly indicated						
d) Limiting use of the hospitals x-ray/ ultrasound						
e)Limiting use of the hospitals CT/MRI machines						
f) Limiting advanced laboratory tests						
g)Screening patient for dialysis						
h)Restricting follow up of NCDs (chronic conditions)						
i)Delaying a treatment or test to see if possible to do without it						
j) Discharging patients earlier than you wanted						
k)Providing second best treatment						
l)Referring patients to other institution						
m) Not informing patients about expensive options						
n)Refuse expensive drugs requested by patients						
o) Others – please specify						

29. Consider a situation when expenses must be covered by the patient. During **the last twelve months**, how often have you saved costs to the **patient/family by**:

	**Daily**	**Weekly**	**Monthly**	**Once in 6 months**	**Never**	**Not applicable**
a)Limiting prescription of brand named drugs						
b)Limiting ward/ICU admission						
c)Limiting surgery unless highly indicated						
d)Limiting screening tests						
e) Limiting x-ray or ultrasound orders						
f)Limiting CT or MRI orders						
g)Limiting advanced lab tests						
h) Screening patient for dialysis						
i)Providing less frequent follow up of NCDs (chronic conditions)						
j)Delaying a treatment or test to see if possible to do without it						
k)Discharging patients earlier than you wanted						
l)Providing second best treatment						
m)Referring patients to other less expensive institution						
n) Not informing the patient about expensive options						
o) Explain costs and benefits of the treatment alternatives for the patient/family and give recommendation on affordable options						
p) Others – please specify						

30. To what extent do you agree or disagree with the following statements.

	Strongly agree	Partly agree	Neutral	Partly disagree	Strongly disagree
a) In my setting, there is lack of enough resources to provide standard medical care					
b)Physicians have the obligation to protect the health care system from avoidable expenses					
C)Costs for the patient is important for me when I decide to use or not to use an intervention					
d) I should sometimes deny beneficial but costly services to certain patients because resources should go to other patients that need them more					
e) Denying medically beneficial but costly services to patients interferes with the doctor-patient relationship					
f) I try to act as my patients advocate to make sure they get the medical services they need					
g) I have seen that health care costs drive people into financial crises					
h) Health care resources in Ethiopia are distributed fairly					
i) Doctors should try to protect poor families from out-of-pocket health expenses, by recommending cheaper, but second best treatment					
j) The financial burden on the health care system is important when I decide to use an intervention or not					
k) I'm given enough freedom to use the unit's resources in my patients' best interest					
l) I find that there is a gap between the recommended medical services and the resources that are currently available in the health care system					
m) In my country there is a gap between what is medically possible and what can be affordable					
n) Physicians should adhere to cost effective standard interventions instead of more expensive interventions that has small proven advantages over the standard intervention					
o) Ongoing and future costs to the patient influence my decisions more than use of hospital resources					
p) If I see that the patient is poor, I do not let the patient know about the expensive option					
q)I refuse some expensive requests from patients					
r)I find that the patients are often forced to pay for diagnostics or treatment that they will not benefit much from in the private clinics					
s) I see examples of patients that are not well informed about the total treatment costs in the private clinics					
t) I see examples of patients that are not well informed about the total treatment costs in the public health care system					

31. One of your patients would benefit from an intervention. This intervention is very expensive. Under these circumstances, which factors/reasons make you more or less likely to use this intervention?

	**Much more likely**	**Somewhat more likely**	**No change**	**Somewhat less likely**	**Much less likely**
a) The patient is old (>75 years)					
b) The patient is a child					
c)The patient is adolescent					
d) The patient is a premature neonate					
e) The patient is poor					
f) The patient has an important position in society					
g)The patient is the only economic provider in the family					
h)The patient is cognitively impaired					
i)The patient is in a prioritized national programs (like HIV, TB)					
j)The patient is a colleague, friend or family					
k) The patient lives far away					
l) The patient urges for the intervention					
m)The intervention has low chance of success					
n) The patient will not work again					
o) The condition requires chronic care					
p)The patient has a rare condition					
q) The condition is attributable to patients unhealthy behaviors like smoking, excessive drinking etc.					
r) The condition is attributable to pregnancy					
s) The intervention is primary prevention					
t) The aim is to improve quality of life in a patient whose life expectancy is short					
u) The aim is to prolong the life of a patient whose quality of life you judge to be low					
v) The benefit to the patient is small					
w) While you think the patient would benefit, the evidence base for the intervention is lacking					
x) The cost of the treatment is covered solely by the government					
y) The cost of the treatment is covered solely by the patient himself					
z) Other (please specify):					

THANK YOU SO MUCH FOR YOUR PARTICIPATION!
